# Dynamic of the Cellular Immune Response at the Dermal Site of *Leishmania (L.) amazonensis* and *Leishmania (V.) braziliensis* Infection in *Sapajus apella* Primate

**DOI:** 10.1155/2014/134236

**Published:** 2014-08-28

**Authors:** Márcia Dalastra Laurenti, Luiz Felipe Domingues Passero, Thaise Yumie Tomokane, Fernanda de Camargo Francesquini, Mussya Cisotto Rocha, Claudia Maria de Castro Gomes, Carlos Eduardo Pereira Corbett, Fernando Tobias Silveira

**Affiliations:** ^1^Infectious Diseases Laboratory (LIM-50), Pathology Department, Medical School of São Paulo University, Avenida Dr. Arnaldo, 455-1^°^ Andar, Sala 1209, 01246-903 São Paulo, SP, Brazil; ^2^Leishmaniasis Laboratory, Evandro Chagas Institute, Ministry of Health, Rodovia BR 316 s/n, 67030-000 Ananindeua, PA, Brazil; ^3^Tropical Medicine Nucleus, Pará Federal University, Avenida Generalíssimo Deodoro 92, 66055-240 Belém, PA, Brazil

## Abstract

The purpose of this study was to characterize the immunopathological response in the skin of *S. apella* infected with *Leishmania (L.) amazonensis* and *L. (V.) braziliensis* parasites, the main causative agents of localized cutaneous leishmaniasis in South America. In infected animals, amastigote forms of *L. (L.) amazonensis* could be detected till 120 days postinfection (PI), while, in *L. (V.) braziliensis* infection, parasites could be detected until 180 days PI in the skin sections. CD20^+^ cells were detected throughout the experimental time in both groups as well as in CD3^+^ cells, which appeared to be activated because high densities of inducible nitric oxide synthase (iNOS^+^) cells were detected at 60 and 90 days PI in both studied groups. After 60 and 120 days PI, decrease in iNOS^+^ cells was observed in *L. (L.) amazonensis* and *L. (V.) braziliensis*, respectively, which was associated with parasite clearance. Increase in lysozyme^+^ cells was observed during the experimental infections, which also can be associated with parasite killing.

## 1. Introduction

Leishmaniasis is an infectious disease caused by different species of protozoa parasites, which is transmitted by sand fly bites. These protozoa are able to cause cutaneous and visceral form of the disease. Several reports have examined the pathogenesis of leishmaniasis in murine models aiming to characterize the mechanisms by which* Leishmania* parasites induce disease [1–5]. However, other reports state that some aspects of leishmaniasis immunopathogenesis cannot be completely represented using murine models since they are not the natural hosts for the parasites. Thus, a more reliable experimental model that mimics human infection is required. Nonhuman primates may represent an interesting tool for analyzing the aspects of human leishmaniasis immunopathology since they share 85–92% of their DNA with humans, indicating their close phylogenetic relationship with humans [6].

The* Sapajus apella *primate, previously known as* Cebus apella*, has been successfully used in experimental studies to analyze the dynamics of clinical and histological lesions during* L.* (*L.*)* amazonensis*,* L.* (*V.*)* braziliensis*, and* L.* (*V.*)* lainsoni* infections [7–9]. In these reports, all species of parasites were able to infect the primates. In addition, animals infected with* L. *(*L.*)* amazonensis *presented an apparent lesion 20 days postinfection (PI) with a nodular aspect recorded at 60 days PI; however, following* L. *(*V.*)* braziliensis *infection, lesions were observed approximately 15 days PI, with spontaneous ulceration verified after 3 months. Histologically, animals infected with* L. *(*L.*)* amazonensis *and* L. *(*V.*)* braziliensis* parasites showed a nonspecific inflammatory infiltrate during the initial phase of infection, characterized by macrophagic nodules, necrosis of inflammatory areas, and the presence of epithelioid granuloma. Absorption of necrotic areas and nonspecific residual inflammatory infiltration with cicatrisation was observed in both groups with disease evolution [9]. Despite the similarities in lesion evolution and in self-healing processes,* L. *(*L.*)* amazonensis*-infected primates presented higher antibody levels than* L. *(*V.*)* braziliensis*-infected primates, suggesting different levels of immune response in both groups that determine the magnitude of disease [10].

Human cutaneous leishmaniasis caused by both of these parasite species showed similar immunopathological features at the dermal site of infection [11–13], supporting the idea that the* S. apella* primate can be used as an experimental model to mimic human disease [7–9]. Studies examining the immunopathogenesis of the* L. (V.) braziliensis* and* L. (L.) amazonensis* infection in humans have not been conclusive, and reports regarding the evolution of infection caused by these parasites species are limited. Thus, shared characteristics among nonhuman primates and humans can aid in the establishment of a very confident experimental model to study American cutaneous leishmaniasis. Since there is little information about the dynamics of cellular immune response in* Leishmania*-infected* S. apella*, the purpose of this study was to analyze the evolution of the immune response developed at the dermal site of* L. *(*V.*)* braziliensis* and* L. *(*L.*)* amazonensis* infection in the neotropical primate* S. apella*.

## 2. Material and Methods

### 2.1. Animals

In this study, we used 10 outbred young specimens of* S. apella* primate, aged 1 to 2 years, weighing between 1,280 and 1,870 g, from both genders, from the National Center of Primates, Ananindeua, PA, Brazil, where they were born from breeding captivity. Before starting the experiments, an indirect fluorescence antibody test (IFAT) and leishmanin skin test (LST) were carried out to exclude the possibility of prior* Leishmania* infection in the animals. The protocol was approved by the Institutional Animals Care and Use of the Evandro Chagas Institute (Ministry of Health, Brazil) and the Animal Care and Use Committee of São Paulo Medical School (0493/07).

### 2.2. Parasites

*L. *(*L.*)* amazonensis *(IFLA/BR/67/PH8) isolated in Belém, PA, and* L. *(*V.*)* braziliensis* (MHOM/BR/88/M11.636) in Monte Dourado, PA, Brazil, were classified by monoclonal antibodies and isoenzymes at the Evandro Chagas Institute, Belém, PA, Brazil.

### 2.3. Experimental Infection

The animals were divided randomly in two experimental groups and then were intradermally infected with 3 × 10^6^ stationary phase promastigotes of* L. *(*L.*)* amazonensis *and* L. *(*V.*)* braziliensis* at six sites of the dorsal surface of the primate tail. Biopsies were collected at 30, 60, 90, 120, 150, and 180 days PI from one of the six sites of infection. Before being biopsied, animals were anesthetized with intramuscular injection of ketamine (20–25 mg/kg) and biopsies were performed using a 4-mm punch. Skin biopsies were fixed in 10% buffered formalin (pH 7.2) and processed by standard histological techniques and immunohistochemistry.

### 2.4. Immunohistochemistry

Briefly, slides with histological sections were deparaffinized and hydrated. Antigenic recovery was developed in citric acid solution (10 mM, pH 6.0) for 3 minutes in a pressure cooker. Next, the slides were washed six times with 3% hydrogen peroxide (H_2_O_2_) to block endogenous peroxidase and to avoid nonspecific ionic binding; the sections were also incubated in a solution of powdered skim milk 10%, diluted in phosphate buffered saline (PBS), pH 7.4 at room temperature for 30 minutes.

The immunolabeling reaction was performed with polyclonal antibodies: mouse anti-*Leishmania* at 1 : 1000 (produced in Laboratory of Pathology of Infectious Diseases) and rabbit anti-human lysozyme at 1 : 800 (A0099, Dako, Carpinteria, CA, USA), and monoclonal antibodies: mouse anti-human CD3 at 1 : 200 (M7254, Dako), rabbit anti-inducible nitric oxide synthase (iNOS) at 1 : 500 (SC-651, Santa Cruz Biotechnology, Santa Cruz, CA, USA), and mouse anti-human CD20 at 1 : 800 (M0755, Dako) diluted in PBS 1% BSA. For development of the reaction, the LSAB kit (Dako) and diaminobenzidine (Sigma, St. Louis, MO, USA) in PBS containing 3% hydrogen peroxide were used. Histological sections were counterstained in Harris's hematoxylin, dehydrated, and mounted in resin with cover slides [14].

At least 10 sequential images of each histological section were acquired using a light microscope equipped with a color video camera connected to computer (Zeiss, Jena, Germany). Immunolabeled cells were quantified by counting in the software AxioVision 4.1 (Zeiss), and cell densities (cells/mm^2^) were calculated. Five biopsies from* S. apella *normal skin were used as negative control.

### 2.5. Statistical Analysis

The results are presented as vertical bars showing the low and high densities of each marker and expressed as the median ± standard error. The parametric test one-way analysis of variance with the Tukey post-test for multiple comparisons was employed to analyze the data. GraphPad Prism version 5 for Windows was used to analyze the results. Differences were considered statistically significant at a 5% significance level (*P* < 0.05).

## 3. Results

### 3.1. Skin Parasitism

**Figure 1:**
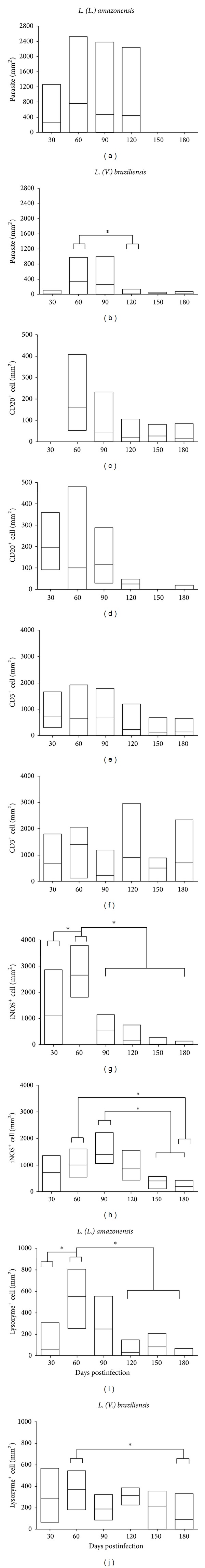
Evolution of parasite and cell densities at the dermal site of* L. *(*L.*)* amazonensis *or* L. *(*V.*)* braziliensis *infection in* S. apella* primates. The bars represent the lowest and highest density of parasitism and the line represents the median. (a) and (b): parasite density; (c) and (d): CD20^+^ cells (B cells) density; (e) and (f): CD3^+^ cells (T cells) density; (g) and (h): iNOS^+^ cell; and (i) and (j): lysozyme^+^ cell. _ _*P < 0.05.

**Figure 2:**
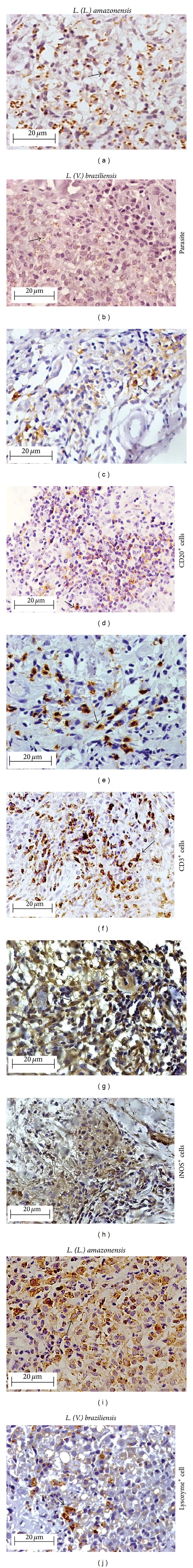
Histological sections of the dermal site of* L. (L.) amazonensis* and* L. (V.) braziliensis* infection at 60 days PI, showing in (a) and (b) amastigotes forms of parasites; (c) and (d) CD20^+^ cells (B cells); (e) and (f) CD3^+^ cells (T cells); (g) and (h) iNOS^+^ cells; and (i) and (j) lysozyme^+^ cells. Immunohistochemistry reaction, Avidin and Biotin System.

Primates infected with* L. *(*L.*)* amazonensis* showed parasites from 30 to 120 days PI with clearance since 150 days PI, when amastigote forms of the parasite could not be detected in the skin histological sections (Figure 1(a)).* L. (V.) braziliensis*-infected primates showed skin parasitism during all the period of the study with a peak between 60 and 90 days PI followed by a decrease since 120 days PI (*P* < 0.05) (Figure 1(b)). Figures 2(a) and 2(b) illustrate amastigote forms in the skin tissue sections of* S. apella* primates infected with* L. (L.) amazonensis *and* L. (V.) braziliensis* at 60 days PI, respectively.

### 3.2. Cellular Immune Response at the Site of Parasite Inoculation

CD20^+^ cells could be detected since 30 days PI in* L. *(*V*.)* braziliensis*-infected primates and in* L. *(*L*.)* amazonensis* since 60 days PI. The density of CD20^+^ cells was similar during the evolution of both infections (Figures 1(c) and 1(d)) with a tendency to decrease with the evolution of the infection. Figures 2(c) and 2(d) illustrate CD20^+^ cells in the skin tissue sections of* S. apella* primates infected with* L. (L.) amazonensis *and* L. (V.) braziliensis* at 60 days PI, respectively.

CD3^+^ cells were observed in the skin of* L. (V.) braziliensis *and* L. (L.) amazonensis *infected primates since 30 days till 180 days PI with no difference during the evolution of the infection (Figures 1(e) and 1(f)). Figures 2(e) and 2(f) illustrate CD3^+^ cells in the skin tissue sections of* S. apella* primates infected with* L. (L.) amazonensis *and* L. (V.) braziliensis* at 60 days PI, respectively.

*L. *(*L.*)* amazonensis*-infected primates showed the presence of iNOS^+^ cells since 30 days PI. The density of iNOS^+^ cells was higher at 60 days PI followed by a significant decrease since 90 days PI (*P* < 0.05) (Figure 1(g)). In the skin biopsies of* Leishmania *(*V.*)* braziliensis-*infected primates, iNOS^+^ cells were also detected since 30 days PI, which gradually increase peaking at 90 days PI, followed by a decrease at 120, 150, and 180 days PI (*P* < 0.05) (Figure 1(h)). Interestingly, the iNOS response occurred earlier in* L. *(*L.*)* amazonensis *infection (60 days PI) than in* L. *(*V.*)* braziliensis *infection (90 days PI), and it was higher in* L. *(*L.*)* amazonensis* infection at 60 days PI (*P* < 0.05). Figures 2(g) and 2(h) illustrate iNOS^+^ cells in the skin tissue sections of* S. apella* primates infected with* L. (L.) amazonensis *and* L. (V.) braziliensis* at 60 days PI, respectively.

Lysozyme^+^ cells were observed since 30 days PI in both experimental groups. A significant increase in* L. *(*L.*)* amazonensis*-infected primates was observed at 60 days PI compared to at 30 days PI (Figure 1(i)), followed by a significant decrease since 90 days PI (*P* < 0.05). In contrast,* L. *(*V.*)* braziliensis-*infected primates showed a similar density of lysozyme^+^ cells till 150 days PI and a significant decrease was verified at 180 days PI compared to at 60 days PI (*P* < 0.05) (Figure 1(j)). Notably, in* L.* (*L.*)* amazonensis* infection, a peaking of lysozyme^+^ cells was observed at 60 days PI and a significant number of stained cells were also observed at 90 days PI compared to* L. *(*V.*)* braziliensis* infection; however,* L. *(*V.*)* braziliensis *infection showed a persistence of lysozyme^+^ cells during the evolution of infection, with a higher density at 120 days PI compared to* L. *(*L.*)* amazonensis* infection (*P* < 0.05). Figures 2(i) and 2(j) illustrate lysozyme^+^ cells in the skin tissue sections of* S. apella* primates infected with* L. (L.) amazonensis *and* L. (V.) braziliensis* at 60 days PI, respectively.

## 4. Discussion

Previous studies showed that* S. apella *primates infected with the* L. *(*L.*)* amazonensis* and* L. *(*V.*)* braziliensis* parasites develop clinical and histopathological skin lesions which spontaneously self-heal and resemble human localized cutaneous lesions [8, 9, 11]. Therefore,* S. apella *primates represent an experimental model of the host resistance since they are able to cure the infection with the parasite elimination.

In this study, the primates infected with* L. *(*L.*)* amazonensis* showed parasites from 30 to 120 days PI, and since 150 days PI no parasites were observed in the tissue. However,* L.* (*V.*)* braziliensis*-infected primates showed skin parasitism during all the period of the study, with a peak between 60 and 90 days PI, followed by a decrease since 120 to 180 days PI. These results show that* L. *(*L.*)* amazonensis* and* L. *(*V.*)* braziliensis* parasites induce, in the* S. apella *primate, a distinct profile of infection, which is closely related to the ability of both parasite species to induce disease and trigger immunity [15].

From the immunopathological perspective, the localized cutaneous leishmaniasis consists of one or more ulcerated skin lesions with the involvement of T and B cells at the lesion site [16]. Together with antigen-presenting cells, B cells participate in the pathogenesis of the lesion by capturing parasite antigens [17] and presenting them to T cells, as well as by antibodies producing, which are not protective to the host [18]. In this study, no significant differences were detected in the densities of CD20^+^ cells during infections caused either by* L. *(*L.*)* amazonensis *or by* L. *(*V.*)* braziliensis*. In contrast, the priming and activation of T cells by antigen-presenting cells are required for promoting self-healing or progression of lesions [19–21]. In both of the experimental groups, T cells were present in similar numbers during the evolution of the infection. Since parasitism was drastically decreased in* L. *(*V.*)* braziliensis *infection at 120 days PI and it was also eliminated in* L. *(*L.*)* amazonensis *infection at 150 days PI, efficient priming and activation of T-cell may have occurred during the course of infection in both infections.

The efficient activation of T cells is important for triggering the production of IFN-*γ*, which activates macrophage cells towards leishmanicidal mechanisms. IFN-*γ*-activated macrophages express iNOS enzyme, which catalyzes nitric oxide production from L-arginine [22]. This immunological pathway is essential for killing intracellular pathogens, such as* Leishmania *sp.; therefore, high expression of the iNOS enzyme is a key requirement for eliminating intracellular amastigotes in the vertebrate hosts [23]. In* L. *(*L.*)* amazonensis*-infected primates, high densities of iNOS^+^ cells were detected mainly at 60 days PI, which probably had an important role in the control and clearance of parasites in the skin of* S. apella *primate, which occur at 150 days PI. In* L. (V.) braziliensis *infection, iNOS^+^ cells reached higher densities between 60 and 90 days PI, however, in lower densities compared to* L. (L.) amazonensis *infection. Interestingly, in* L. (V.) braziliensis *infection, few parasites persisted in the skin until the end of the experimental time, which could be a direct result of the lower number of iNOS^+^ cells presented in this group. In addition, other mechanisms may collaborate to the parasite control and clearance, such as those based on lysozyme production.

Lysozyme is an antimicrobial peptide that is part of the innate immune response and can be produced by neutrophils, monocytes, and macrophages [24] and it appears to participate in microbial mechanisms for parasite destruction [25]. In* L. *(*L.*)* amazonensis-*infected primates, increase in the density of lysozyme^+^ cells was directly correlated with the increase in the number of iNOS^+^ cells at 60 days PI, suggesting that parasite elimination could have occurred by both of these activation mechanisms. In contrast, in* L. *(*V.*)* braziliensis*-infected primates, the density of lysozyme^+^ cells was similar throughout the infection, and at 90 and 120 days PI it was lower than that observed in primates infected with* L. *(*L.*)* amazonensis*, indicating that parasite destruction occurs predominantly through mechanisms involving nitric oxide. Lysozyme may play a role in parasite destruction; however, it participates to a lower extent than nitric oxide in both groups since the densities of lysozyme^+^ cells were lower than those of iNOS^+^ cells in the inflammatory infiltrate of* S. apella* skin.

## 5. Conclusion 

The present results indicate that* S. apella *primates are interesting models to evaluate immunological parameters related to self-healing in localized cutaneous leishmaniasis, caused by both* L. (L.) amazonensis* and* L. (V.) braziliensis*. These results indicate that the resistance against the infection can be triggered by components of both innate and acquired immunity. Differences in parasite species and its immunomodulatory properties on the vertebrate host cells impact the outcome of infection, as observed in the evolution of the infection caused by* L. *(*L.*)* amazonensis *and* L. *(*V*.)* braziliensis* in* S. apella *primate.
